# Shift from income breeding to capital breeding with latitude in the invasive Asian shore crab *Hemigrapsus sanguineus*

**DOI:** 10.1038/s41598-024-57434-y

**Published:** 2024-03-20

**Authors:** Tanner C. Reese, April M. H. Blakeslee, Laura C. Crane, Laura S. Fletcher, Michele F. Repetto, Nanette Smith, Carter Stancil, Carolyn K. Tepolt, Benjamin J. Toscano, Blaine D. Griffen

**Affiliations:** 1 Department of Biology, Brigham Young University, Provo, UT 84602 USA; 2 Department of Biology, East Carolina University, Greenville, NC 27858 USA; 3 Wells National Estuarine Research Reserve, Wells, ME 04090 USA; 4 Department of Biology, Temple University, Philadelphia, PA 19122 USA; 5 Department of Biology, Woods Hole Oceanographic Institution, Woods Hole, MA 02543 USA; 6 Department of Biology, Trinity College, Hartford, CT 06106 USA

**Keywords:** Ecophysiology, Animal physiology

## Abstract

Organisms vary in the timing of energy acquisition and use for reproduction. Thus, breeding strategies exist on a continuum, from capital breeding to income breeding. Capital breeders acquire and store energy for breeding before the start of the reproductive season, while income breeders finance reproduction using energy acquired during the reproductive season. Latitude and its associated environmental drivers are expected to heavily influence breeding strategy, potentially leading to latitudinal variation in breeding strategies within a single species. We examined the breeding strategy of the Asian shore crab *Hemigrapsus sanguineus* at five sites spanning nearly 10° of latitude across its invaded United States range. We hypothesized that the primary breeding strategy of this species would shift from income breeding to capital breeding as latitude increases. We found that though this species’ breeding strategy is dominated by capital breeding throughout much of the range, income breeding increases in importance at lower latitudes. This latitudinal pattern is likely heavily influenced by the duration of the foraging and breeding seasons, which also vary with latitude. We also found that reproductive characteristics at the northern and southern edges of the invaded range were consistent with continued range expansion. We suggest that the reproductive flexibility of the Asian shore crab is a key facilitator of its continued invasion success. Our results highlight the influence of latitude on the breeding strategy of a species and emphasize the need for further research regarding the ecological importance and implications of flexibility in breeding strategies within species.

## Introduction

Energetically financing reproduction is a fundamental biological process of all organisms. There is, however, considerable variation among organisms and taxa in the extent to which energy acquisition for reproduction and reproduction itself occur as discrete events or as continual processes^1^. The timing of energy acquisition and allocation to reproduction, relative to the timing of energy use in reproduction, depends largely on the availability of resources^2^ and on the length of the reproductive season^3^. Two broad strategies, capital breeding and income breeding, have been defined as the endpoints of the breeding strategy continuum, with perhaps the majority of species falling somewhere between these two extremes^4,5^. Here, we define capital breeding as reproduction that uses energy acquired and stored prior to the start of the reproductive period. This strategy is often observed in species that have limited access to food during the breeding season or that experience long periods of fasting, such as baleen whales and phocid pinnipeds^6^. We define income breeding as when organisms finance offspring production using energy acquired during the reproductive period^1^. This strategy is common in species that have abundant food resources throughout the breeding season, such as odontocete whales and otariid pinnipeds^6^.

An organism’s breeding strategy plays a central role in the ecology of systems. For instance, seabird spatial ecology is heavily influenced by breeding strategy and breeding energetics, with capital breeding most common in migratory species that travel between feeding grounds and breeding grounds at different times of the year^7^. However, some migratory seabird species, such as the greater snow goose (*Chen caerulescens atlantica*), utilize elements of both capital and income breeding strategies, suggesting that not all migratory species fall neatly into these categories^8^. The breeding strategy, or combination of breeding strategies, used by a species has significant effects on the seasonality of the species’ energetic requirements, and thus on its diet and resource use. In turn, resource acquisition impacts interactions with other species in its environment through the timing of trophic interactions and resultant competition for trophic resources. Breeding strategy further influences the ability of a species to withstand environmental change—for example, periods of decline in habitat quality may overlap differently with the periods for energy acquisition in capital and income breeders, thus differentially affecting reproductive success and population stability^9^.

The use of capital versus income breeding strategies is expected to differ with the relative length of the foraging season, the length of the reproductive season, and the number of reproductive bouts that are attempted in a reproductive season. It has been hypothesized that in high latitude regions where foraging periods are short and intense, and young have a relatively brief period of time to become established before the winter dormant period, organisms should primarily use a capital breeding strategy^10^. In this case, energy acquired during the summer and autumn foraging season in one year can be stored and used as capital to produce offspring early in the following spring. This strategy gives offspring sufficient time to establish and develop throughout the summer before winter conditions set in. This general high-latitude theoretical scenario was empirically supported in the copepod *Calanoides acutus*^10^. This study also found that additional offspring of lower quality could be produced later that same year via income breeding, using energy acquired during that year’s foraging^10^. Though later and lower-quality offspring are less likely to establish before winter and survive, some offspring may survive and thus contribute to the overall biological fitness of the parent^11^. In contrast to this strategy used by some organisms at high latitudes, organisms at lower latitudes that experience reduced seasonality (i.e., those in tropical and subtropical environments) should have a less discrete foraging season and therefore continually acquire energy throughout the year using an income breeding strategy^3^. The reproductive season should also be extended at these lower latitudes and in the extreme case of the tropics, may span the entire year.

The use of a capital or an income breeding strategy likely is not ‘either-or’, but instead represents endpoints of a continuum^5^. It is possible therefore that species with broad geographic ranges may shift along this continuum throughout their latitudinal range as environmental drivers change. Such species would not be characterized as solely capital or income breeders, but as species with a flexible breeding strategy that varies based on latitudinally-dependent environmental factors. Though some studies have examined how reproductive strategies and characteristics vary with latitude^12–15^, to our knowledge only three studies have empirically examined shifts along the capital/income breeding continuum for a single species^3,10,16^. Each of these studies investigated shifts from capital to income strategies through the reproductive season at a single site, but did not consider shifts along a latitudinal gradient. Understanding how reproduction varies across latitude is particularly important as species continue to shift their ranges latitudinally due to climate change and human-mediated dispersal.

Crabs are a useful study organism for exploring the capital to income breeding continuum^3,9^ because they store energy long-term via lipids synthesized and stored in the hepatopancreas^17^, a digestive organ, and these energy reserves can be quantified gravimetrically. This facilitates accurate assessment of energy allocation to reproduction, as stored energy is transferred from the hepatopancreas to the ovary for use in egg production^18^. Historically, crabs have been considered as capital breeders^19^, but more recently have been demonstrated to use both capital and income strategies^9^. Additionally, some crabs employ an intermediate/mixed breeding strategy, using primarily stored capital to produce eggs during the early part of the reproductive season before shifting to income breeding for later egg production once that stored capital is expended^3,16^.

We investigated breeding strategies across latitude in introduced populations of the Asian shore crab *Hemigrapsus sanguineus.* Native to the western Pacific from Hong Kong to southern Russia, this species has invaded intertidal regions of both North America and Europe^20^. The Asian shore crab was first documented in North America in Cape May, NJ in 1989^21^. This species now spans a large invasive range from North Carolina to Maine^20^, with some individuals recently reported in Canada^22^.

Asian shore crabs produce multiple clutches of eggs annually^23^, though their breeding strategy remains uncertain^24^, possibly because this species may not fit neatly within a dichotomous breeding perspective. This species occurs over a broad geographic range and therefore environmental conditions differ considerably across populations. In northern regions of its North American invaded range (within New England), environmental conditions are highly seasonal, with relatively long winter periods of inactivity. In southern regions (New Jersey, Virginia, North Carolina), environmental conditions are less extreme and individuals have much shorter winter inactive periods.

Here, we tested the hypothesis that female Asian shore crabs employ a primarily capital breeding strategy in the northern part of their North American range and a primarily income breeding strategy in the southern part of this range, with an intermediate breeding strategy in the central regions. To test our hypothesis we sampled crabs from five locations spread relatively evenly throughout the invaded range, sampling at each location throughout an entire reproductive season. We measured ovary, egg, and hepatopancreas mass from each sampling location to test our hypothesis by comparing observed patterns to indicators of capital and income breeding. Tissue mass was used as a valid proxy for tissue energy because the masses of these tissues are primarily lipids that were originally synthesized in the hepatopancreas before being transferred to other tissues^17^. We test our general hypothesis by examining five indicators of whether capital or income breeding is being used (summarized in Table 1).

**Table 1:** Expected and observed patterns used as indicators of capital and income breeding.

Metric (all measured throughout the reproductive period)	Indicates capital breeding	Indicates income breeding	Observed pattern
1. Ovary mass^3^	Decrease	Increase or no change	ME:↓; NH:↓; CT:↓; NJ:↓; NC:↓
2. Egg mass^3^	Decrease	Increase	ME:↓; NH: − ; CT:↓; NJ:↓; NC:↓
	No change could support either capital or income breeding		
3. Hepatopancreas mass^3^	Decrease	Increase or no change	ME:↓; NH:↑; CT: − ; NJ:↓; NC: −
4. Mass transfer between hepatopancreas (HSI) and ovary (GSI)^19^	Inverse correlation between HSI and GSI	No correlation between HSI and GSI	Inverse correlation between HSI and GSI (data from all sites were combined)
5. Pre-reproduction energy storage (SM: energy contained in the hepatopancreas and ovary, as measured by mass) compared to average mass of first clutch (EM)^3^	SM > EM	SM < EM	ME: SM > EM
			NH: SM > EM
			CT: SM = EM
			NJ: SM < EM
			NC: SM < EM

↓ = decrease; −  = no change; ↑ = increase. SM = storage mass; EM = egg mass; ME = Maine; NH = New Hampshire; CT = Connecticut; NJ = New Jersey; NC = North Carolina. “Northern regions” referred to in the text are comprised of ME and NH, “southern regions” are comprised of NJ and NC, and the “central” region is CT.

Indicators 1–3 (Table 1): decreased ovary, egg, or hepatopancreas mass throughout the reproductive season indicates capital breeding as stored energy is depleted by reproduction. Conversely, no change (or an increase) in ovary or hepatopancreas mass throughout the reproductive season suggests income breeding, while no change in egg mass throughout the reproductive season provides no information on the source of energy for eggs and therefore does not distinguish between capital or income breeding. Indicator 4 (Table 1): an inverse correlation between hepatopancreas and ovary mass indicates capital breeding as lipids are transferred from the hepatopancreas to the ovary for egg production. Indicator 5 (Table 1): if the mass of lipids stored in the ovary + hepatopancreas before reproduction begins exceeds the mass of the first egg clutch, then capital breeding is possible; whereas if the egg mass of the first clutch exceeds the combined mass of these two organs, then income breeding is necessary to finance even the first clutch of eggs.

## Results

### Ovary mass

**Figure 1:**
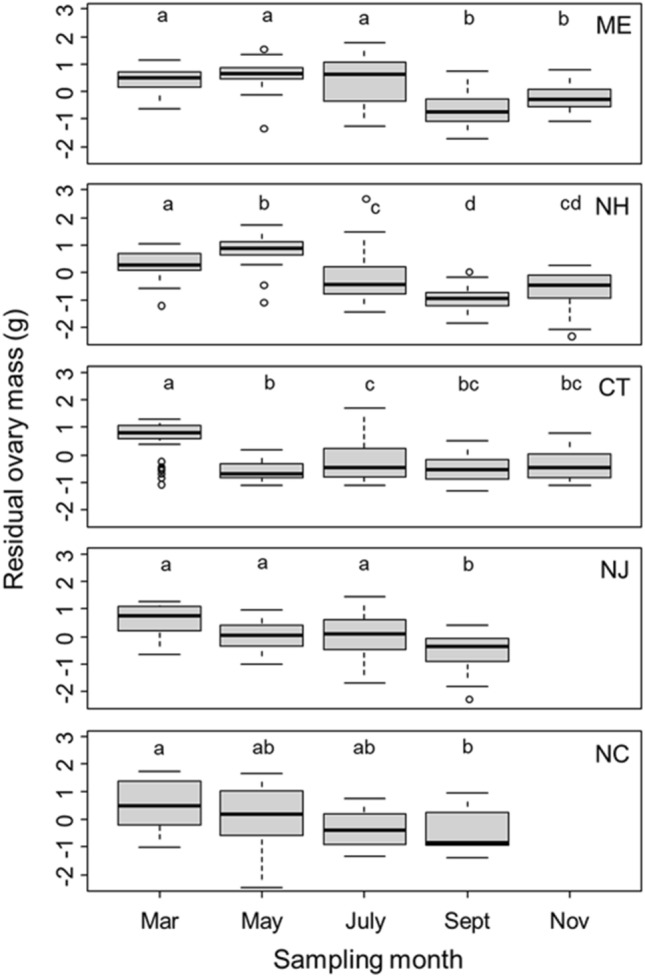
Residual ovary mass of *Hemigrapsus sanguineus* across months for each of the five sampling sites, indicated by the state abbreviation in the upper right corner of each plot. Heavy black line shows median value, boxes encompass the interquartile range (i.e., 25th–75th percent of the data), whiskers encompass 1.5 × the interquartile range, and circles represent data points that fall outside that range. Letters over the boxplots show statistical significance within each site only (from site-specific ANOVAs and Tukey’s tests), where boxplots with the same letter are not significantly different.

**Figure 2:**
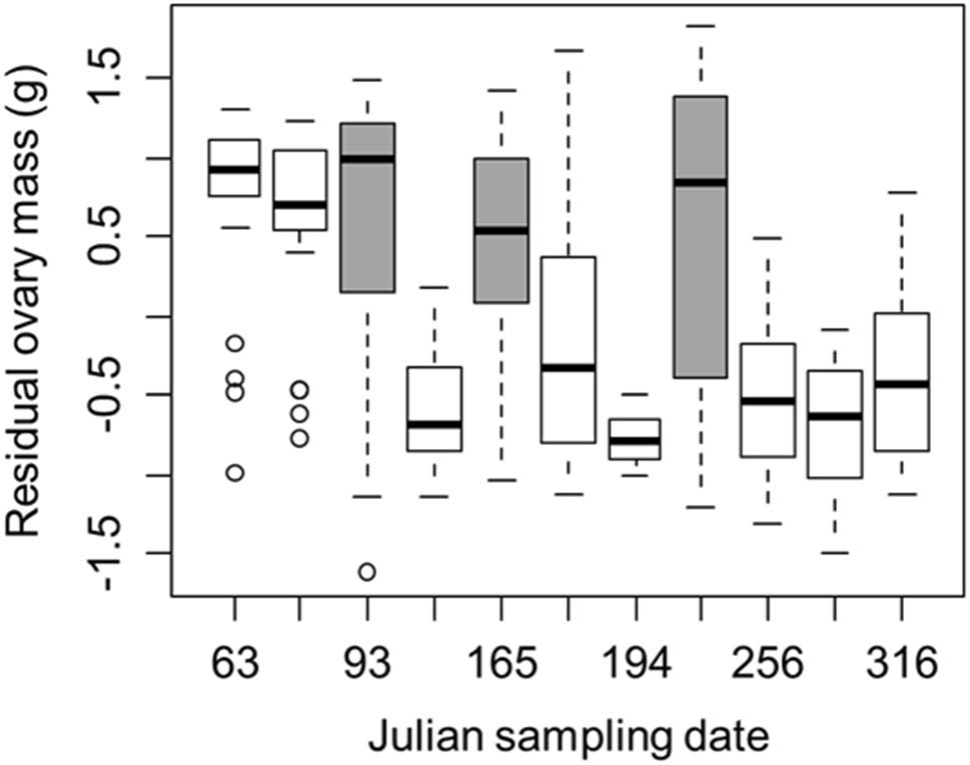
Residual ovary mass of *Hemigrapsus sanguineus* at the Connecticut sampling site where sampling occurred more frequently. The data show three primary reproductive peaks representing three distinct clutches occurring up to Julian sampling date 93 and around sampling dates 165 and 226. The boxplots corresponding to these primary reproductive peaks are colored gray. Boxplots are as described in the caption for Fig. 1.

We found that residual ovary mass differed across sites (*F*_4_ = 5.74, *P* < 0.001) and varied with sampling month (*F*_4_ = 61.94, *P* < 0.001). Further, we found a significant interaction between sampling site and sampling month (*F*_14_ = 7.67, *P* < 0.001). Using site-specific ANOVAs, we found that residual ovary mass decreased throughout the summer reproductive season at all sites (ME: *F*_4_ = 19.35, *P* < 0.001; NH: *F*_4_ = 46.26, *P* < 0.001; CT: *F*_4_ = 30.00, *P* < 0.001; NJ: *F*_3_ = 10.05, *P* < 0.001; NC: *F*_3_ = 3.38, *P* = 0.024; indicator 1 in Table 1 and Fig. 1). Thus, there was no observable shift in ovary dynamics with latitude. When residual ovary mass was examined through time at only the Connecticut site (i.e. the central site), which had more frequent sampling than the other sites, the 6th order polynomial provided the best fit to the data, with lower order polynomials providing progressively poorer fit (ΔAIC ≥ 3.02), suggesting three primary reproductive peaks near Julian sampling dates 93 (April 3), 165 (June 14), and 226 (August 14; Fig. 2). These reproductive peaks represent three distinct peaks in clutch production at the Connecticut site (see gray-colored boxplots in Fig. 2). Sampling at sites other than Connecticut was not sufficiently frequent to determine the number of clutches produced at those sites.

### Egg mass

**Figure 3:**
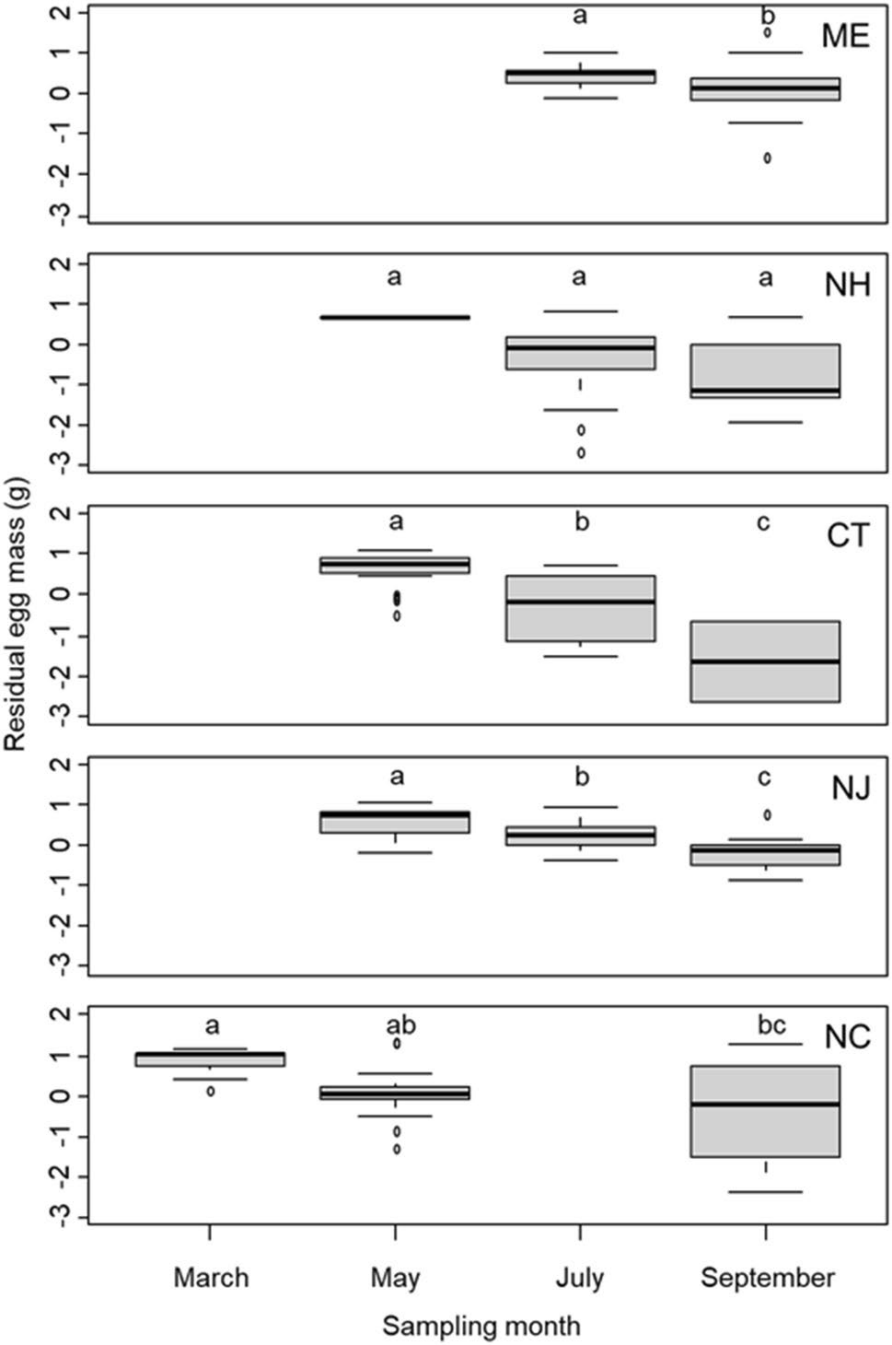
Residual egg mass of *Hemigrapsus sanguineus* across months for each of the five sampling sites, indicated by the state abbreviation in the upper right corner of each plot. Boxplots and letters over the boxplots are as described in the caption for Fig. 1.

We found that residual egg mass differed across sites (*F*_4_ = 16.26, *P* < 0.001), was highest toward the northern and southern latitudes (i.e., at the edges of this species’ invaded range), and varied with sampling month (*F*_5_ = 72.64, *P* < 0.001). Further, there was a significant interaction between sampling site and sampling month (*F*_6_ = 3.07, *P* = 0.017). We also found that throughout the summer reproductive season, residual egg mass decreased at four sites (ME: *F*_1_ = 4.44, *P* = 0.040; CT: *F*_4_ = 24.57, *P* < 0.001; NJ: *F*_2_ = 20.92, *P* < 0.001; NC: *F*_2_ = 5.05, *P* = 0.013) and did not change significantly at one site (NH: *F*_2_ = 1.27 *P* = 0.291; indicator 2 in Table 1 and Fig. 3). When residual egg mass was examined through time at only the Connecticut site (with a linear model), residual egg mass also decreased throughout the summer reproductive season (*t* = − 7.16, *P* < 0.001).

### Hepatopancreas mass

We found that residual hepatopancreas mass differed across sites (*F*_4_ = 3.51, *P* = 0.008) and varied with sampling month (*F*_4,616_ = 3.39, *P* = 0.009). Further, we found a significant interaction between sampling site and sampling month (*F*_14,616_ = 9.85, *P* < 0.001). Based on individual site ANOVAs, we found that residual hepatopancreas mass decreased throughout the summer reproductive season at two sites (ME: *F*_4_ = − 2.97, *P* = 0.022; NJ: *F*_3_ = 42.65, *P* < 0.001), increased at one site (NH: *F*_4_ = 9.96, *P* < 0.001), and did not change at two sites (CT: *F*_4_ = 1.25, *P* = 0.293; NC: *F*_3_ = 0.50, *P* = 0.684; indicator 3 in Table 1). These differences across sites therefore showed no clear latitudinal trend. When examined through time at only the Connecticut site, residual hepatopancreas mass varied cyclically with time (first order term, *t* = 1.90, *P* = 0.059; second order term, *t* = − 1.92, *P* = 0.056; third order term, *t* = 1.96, *P* = 0.051; fourth order term, *t* = − 2.03, *P* = 0.044; fifth order term, *t* = 2.11, *P* = 0.036, sixth order term, *t* = − 2.21, *P* = 0.023), consistent with lipid transfer from the hepatopancreas to the ovary for egg production.

### Mass transfer between hepatopancreas (HSI) and ovary (GSI)

When the data from all sites were analyzed together, we found that the hepatosomatic index decreased as the gonadosomatic index increased (*t* = − 2.13, *P* = 0.034; indicator 4 in Table 1), again consistent with a shift of stored energy from the hepatopancreas to the ovaries.

### Pre-reproduction storage compared to mass of first clutch

**Figure 4:**
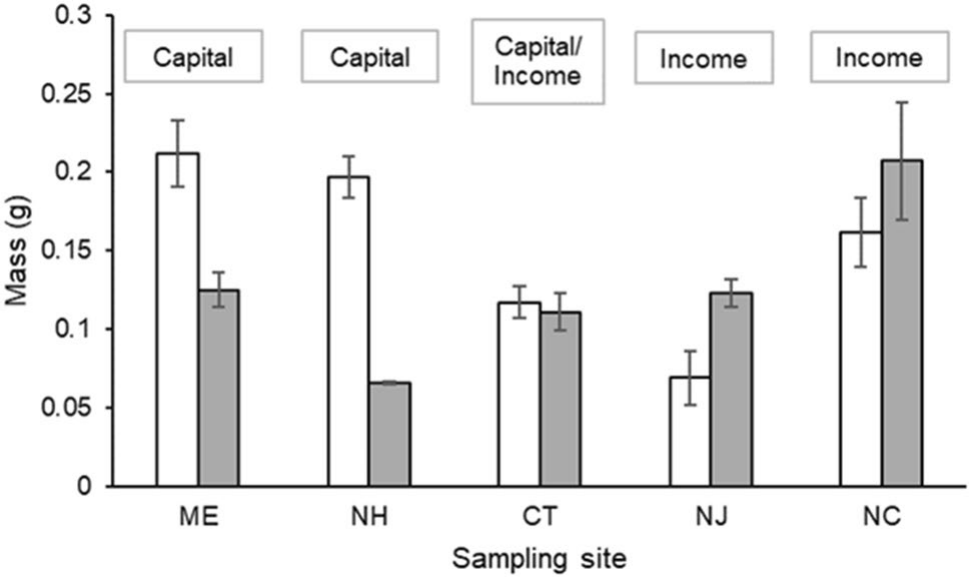
Comparison of pre-reproduction energy storage (energy contained in the hepatopancreas and ovary, as measured by mass; white bars) to egg mass of first clutch (gray bars) in *Hemigrapsus sanguineus* indicates which breeding strategy is dominant at a site, where storage mass > egg mass is consistent with capital breeding, and storage mass < egg mass indicates income breeding. Error bars show one standard error from the mean. Gray boxes above each pair of bars indicate the predominant breeding strategy at that site. Sampling locations are listed along a decreasing latitudinal gradient from left to right.

The test of indicator 5 showed the clearest evidence of a latitudinal trend in reproductive strategy. Specifically, we found evidence of a latitudinal cline in the relative mass of pre-reproduction energy storage (energy contained in the hepatopancreas and ovary, as measured by mass) and the average mass of the first clutch of eggs. Pre-reproduction stored energy mass was greater than the mass of the first egg clutch at the two northern sites, was similar to the mass of the first egg clutch at the central Connecticut site, and was less than the mass of the first egg clutch at the two southern sites (indicator 5 in Table 1 and Fig. 4).

## Discussion

We have shown that the Asian shore crab uses a mixed breeding strategy that appears to be dominated by capital breeding throughout much of the range (indicators 1–4 in Table 1). However, income breeding increases in importance at lower latitudes (indicator 5 in Table 1). Additionally, we have shown that residual egg mass is highest toward the northern and southern edges of this species’ invasive range, consistent with patterns expected for a species continuing to expand its range^25^. Below, we discuss general patterns and implications that can be drawn from our results and evaluate our results in the context of our study system.

Our main finding provides support for the theory put forth by Varpe et al.^10^, namely that organisms at high latitudes should primarily employ a capital breeding strategy. This pattern has important ecological implications across a variety of systems. For instance, short-term pulses in environmental conditions may be less likely to impact the reproduction of a population at higher latitudes of its range, if at these latitudes the population is primarily employing capital breeding and therefore relying more on stored energy obtained over a short period of time^2^. At lower latitudes, a population of the same species is more likely to employ an income breeding strategy. It may therefore be more likely to be affected by short-term variation in environmental conditions, as foraging occurs over a much longer timescale and is thus more likely to overlap with environmental disturbances. The carryover effects experienced by a species—when processes or events in one season influence the performance of individuals in the following season—may also be impacted by this pattern of latitudinal variation in breeding strategy^26^. Our results predict that carryover effects should be stronger at higher latitudes because primarily capital-breeding populations (i.e., populations at higher latitudes) store energy during a foraging season that will be used in a subsequent reproductive season. For high-latitude populations of our study species, foraging in the late summer and autumn likely finances reproduction in the following spring. This pattern appears to be common in seasonal environments occurring across a wide range of animals, including mule deer^27^, rattlesnakes_^28^, and copepods^29^.

High-latitude reliance on capital breeding results from the combined influence of short foraging and short reproductive seasons. For many organisms, the foraging season is generally shorter at higher latitudes due to colder winter temperatures that decrease movement and feeding^30^. Many crab species at high latitudes, such as Tanner crabs (*Chionoecetes bairdi*)^31^ and harbour crabs (*Liocarcinus depurator*)^32^, produce only one or two clutches of eggs annually due to the shortened season in which females can obtain and store energy for egg production. Because clutches beyond the first clutch are less likely to be produced—especially at higher latitudes where food availability is less consistent^30^—and the first clutch is likely produced using some stored capital^12^, it is expected that the first clutch of eggs will be larger and contain more energy than subsequent clutches. Our findings that the residual mass of egg clutches decreased throughout the breeding season at almost all sites support this idea. The single site with no significant decrease in egg clutch mass throughout the season (NH) likely reflects a small sample size and insufficient statistical power for detecting an effect.

At lower latitudes, animals often produce multiple clutches of eggs during their breeding season^33–35^, though the first clutch is often the highest quality^36^ for reasons outlined above. Yet even primarily income-breeding females, which are more common at lower latitudes (Fig. 4), likely store up some energy late in the foraging season (i.e., capital breeding) that can contribute to the first clutch of eggs produced during the next breeding season^10^, increasing the quality and size of the first clutch. This reasoning is supported by both our results that residual ovary mass in Asian shore crabs decreased throughout the summer reproductive season at all latitudes as crabs depleted whatever amount of energy they had stored, and by the life history model developed by Varpe et al.^10^ for copepods.

Ectothermy may also play a role in this observed latitudinal breeding pattern. Ectotherms at lower latitudes have higher metabolic expenditures throughout the year because of warmer average temperatures, and also have higher metabolic costs of energy storage^37^. Thus, for a given level of food consumption, organisms at lower latitudes have reduced capacity to store energy because they are burning more energy for basal metabolism and maintenance, necessitating the use of income breeding to acquire sufficient energy for egg production. However, the effects of this negative relationship between temperature and energy storage are potentially tempered by increased foraging activity at warmer temperatures^38,39^.

In addition to the patterns of reproductive energetics examined here, our study also has implications for the continued range expansion of Asian shore crabs and similar species. We found that residual egg mass was highest towards the edges of this species’ invaded range, which is consistent with the expectations for a range-expanding species because populations at the range edge are less dense and thus experience reduced competition for resources^25^. The Asian shore crab is a highly successful invader in many different habitats and boasts a large latitudinal range^20^. Much research has examined the factors that make an invasive species successful^24,40–42^, though many questions remain. Our results point to reproductive flexibility as an important factor in improving a species’ invasion success, particularly when that invasion occurs over a wide range of environments and latitudes.

In summary, the breeding strategy of the Asian shore crab varies with latitude, from being capital breeding dominant at higher latitudes to income breeding dominant at lower latitudes. Thus, the Asian shore crab cannot be defined solely as a capital or income breeder. Which breeding strategy dominates at a particular latitude is likely influenced by several interacting factors, including the lengths of the foraging and reproductive seasons that are determined by air and water temperatures. This reproductive flexibility may contribute to the highly successful invasion of the Asian shore crab and play a key role in the success of other species with broad geographic ranges that span variable latitudes. These results underscore the need for further study of breeding flexibility in other systems and the role that this plays in species’ responses to continued environmental change.

## Materials and methods

### Crab collection and sampling sites

We hand-collected 799 mature female Asian shore crabs from March 1 to November 11, 2020 from five sites along the Atlantic Coast of the United States. Collected crabs were frozen and shipped on dry ice to Brigham Young University in Provo, UT and stored at − 80 °C until they were dissected.

The five sampling sites included: 1. Bailey Island in Harpswell, Maine (ME); 2. Odiorne State Park in Rye, New Hampshire (NH); 3. Goshen Point at Harkness Memorial State Park in Waterford, Connecticut (CT); 4. Cape May Ferry, North Cape May, New Jersey (NJ); and 5. Oregon Inlet, North Carolina (NC). Sites were spread relatively evenly from north to south over the majority of the established North American range of the Asian shore crab. For a more detailed description of the physical characteristics of each site, see Reese et al.^43^ which used the guts of these same specimens in a separate study. At the Maine, New Hampshire, New Jersey, and North Carolina sites, sampling occurred every other month. At the Connecticut site, sampling occurred every month to allow temporal differences between study variables to be resolved on a finer scale at a single site. Sample sizes at each collection period are given in Table 2.

**Table 2:** Sampling site coordinates and site-specific sampling dates and sample sizes.

Site (region)	Latitude and longitude	Julian sampling dates (sampling size)
Maine (northern)	43° 43′ 2.7336″ N, − 70° 0′ 11.4624″ W	75 (20), 133 (30), 192 (41), 252 (34), 314 (32)
New Hampshire (northern)	43° 2′ 20″ N, − 70° 42′ 55″ W	61 (24), 133 (32), 194 (33), 251 (36), 313 (32)
Connecticut (central)	41° 17′ 56.1″ N, 72° 06′ 44.9″ W	63 (21), 75 (27), 93 (33), 136 (30), 165 (30), 181 (30), 194 (3), 226 (30), 256 (28), 285 (26), 316 (30)
New Jersey (southern)	38° 58′ 3.396″ N, − 74° 57′ 45.9858″ W	66 (18), 131 (35), 193 (35), 258 (33)
North Carolina (southern)	35° 46′ 7.33″ N, 75° 31′ 37.76″ W	75 (27), 136 (30), 197 (5), 259 (14)

### Sample processing

Frozen crabs were thawed in water to room temperature and dissected using dorsal carapace removal. The ovaries, eggs, hepatopancreas, and remaining body parts of each crab were separated and placed in separate aluminum weigh boats and dried to constant weight at 60 °C (e.g.^43^). Once dry, we weighed each tissue to the nearest 0.01 mg using a XS205DU Mettler Toledo semimicro balance. Internal eggs (i.e., from vitellogenic crabs) were counted as part of the ovary mass.

### Analyses

#### Residual tissue mass

We examined changes in ovary, egg, and hepatopancreas mass through time to test whether the mass of these tissues decreased on average over the course of the reproductive season, a trend consistent with capital breeding (Table 1). We used identical statistical approaches to analyze each of these tissues. First, we regressed the log of each of these metrics individually on log crab body mass and then used the residuals from these analyses as the response variables in further analyses in order to control for effects of body size. We ran ANOVAs with sampling month, sampling site, and their interaction as predictor variables. If the interaction was significant, we ran site-specific ANOVAs with sampling month as the predictor variable and residual tissue mass as the response variable. Each site-specific ANOVA was followed by a Tukey’s HSD test to compare residual tissue mass between the sampling dates.

More frequent sampling at the Connecticut site allowed for greater detection of temporal patterns. We therefore fit models using Connecticut data only with residual ovary and residual hepatopancreas mass as the response variables and first, second, third, fourth, fifth, and sixth order polynomials of Julian sampling date (i.e., day of the year) as the predictor variables (highest order based on visual inspection of the data; Fig. 2). We then compared these six models for each response variable using AIC to select the best-fitting model for each tissue. This analysis with residual ovary mass allowed us to detect reproductive peaks associated with peaks in clutch production and to examine indicator 1 (Table 1). Residual hepatopancreas mass was analyzed in this way to maintain consistency in our statistical analyses and to further test our hypothesis that tissue mass, a proxy for stored energy, would decrease through time (indicator 3; Table 1). Because only five sampling dates from the Connecticut site had eggs and no nonlinear trends through time were evident, we examined how egg mass changed through time by fitting a single linear model with Julian sampling date as the predictor variable and residual egg mass as the response variable. This analysis was performed to test indicator 2 (Table 1).

#### Mass transfer between hepatopancreas (HSI) and ovary (GSI)

A linear model was used to explore the relationship between ovary mass and hepatopancreas mass, with the gonadosomatic index (defined as a crab’s ovary mass divided by its body mass and then multiplied by 100, for scaling purposes) as the predictor variable, and the hepatosomatic index (defined as a crab’s hepatopancreas mass divided by its body mass and then multiplied by 100) as the response variable. This analysis was conducted using combined data from all sites (to maximize statistical power) and tested indicator 4 (Table 1).

#### Pre-reproduction storage compared to mass of first clutch

To determine whether breeding strategy changes with latitude, we qualitatively compared the ratio of each site’s mean pre-reproduction energy storage (combined mass of the ovary and hepatopancreas) to the mean mass of the first egg clutch at that same site to determine whether energy stores before the start of the reproductive season were sufficient to account for initial reproductive efforts. A high ratio (storage mass/clutch mass) indicates that capital breeding is likely and a low ratio indicates that income breeding is necessary. This analysis tested indicator 5 (Table 1). All statistical tests were performed using R statistical software^44^.

## Data Availability

The datasets generated during and/or analyzed during the current study are available from the corresponding author on reasonable request.
